# The *Drosophila* homologue of Rootletin is required for mechanosensory function and ciliary rootlet formation in chordotonal sensory neurons

**DOI:** 10.1186/s13630-015-0018-9

**Published:** 2015-07-02

**Authors:** Katarzyna Styczynska-Soczka, Andrew P. Jarman

**Affiliations:** Centre for Integrative Physiology, University of Edinburgh, Edinburgh, EH8 9XD UK

**Keywords:** Rootlet, Rootletin, *Drosophila*, Centrosome, Basal body, Sensory cilium

## Abstract

**Background:**

In vertebrates, rootletin is the major structural component of the ciliary rootlet and is also part of the tether linking the centrioles of the centrosome. Various functions have been ascribed to the rootlet, including maintenance of ciliary integrity through anchoring and facilitation of transport to the cilium or at the base of the cilium. In *Drosophila*, *Rootletin* function has not been explored.

**Results:**

In the *Drosophila* embryo, *Rootletin* is expressed exclusively in cell lineages of type I sensory neurons, the only somatic cells bearing a cilium. Expression is strongest in mechanosensory chordotonal neurons. Knock-down of *Rootletin* results in loss of ciliary rootlet in these neurons and severe disruption of their sensory function. However, the sensory cilium appears largely normal in structure and in localisation of proteins suggesting no strong defect in ciliogenesis. No evidence was found for a defect in cell division.

**Conclusions:**

The role of Rootletin as a component of the ciliary rootlet is conserved in *Drosophila*. In contrast, lack of a general role in cell division is consistent with lack of centriole tethering during the centrosome cycle in *Drosophila*. Although our evidence is consistent with an anchoring role for the rootlet, severe loss of mechanosensory function of chordotonal (Ch) neurons upon *Rootletin* knock-down may suggest a direct role for the rootlet in the mechanotransduction mechanism itself.

## Background

The ciliary rootlet has long been known from transmission electron microscopy studies as the striated fibrous structure extending from the cilium basal body towards the cell nucleus [1]. A rootlet is present at the base of most cilia, but it is particularly robust in cells with large or motile cilia. For instance, mammalian photoreceptors have a large rootlet at the base of a connecting cilium that links to the large photoreceptive outer segment [2, 3]. The rootlet has been speculated to have various functions, including contraction, association with organelles, transport/trafficking and anchoring of the basal body and axoneme [1].

Knowledge of the rootlet was advanced by the discovery of its major constituent protein, a coiled-coil protein known as rootletin (encoded by the *CROCC* [ciliary rootlet coiled-coil] gene in humans) [4]. Mouse rootletin is a large 2009 amino acid residue protein with a globular head domain and a tail domain consisting of extended coiled-coil structures [4]. The tail domain mediates polymerisation, whilst the head domain interacts with kinesin light chain 3 (KLC-3) [5]. It seems likely that rootletin is the only structural constituent of the ciliary rootlet, and its depletion causes loss of the rootlet [6, 7]. Hence, rootletin-deficient mice have been used to assess the function of the rootlet. Interestingly, mice lacking rootletin only exhibit a prominent ciliary phenotype in photoreceptors [6], which are cells with high rootletin expression levels and a particularly robust rootlet [2, 3]. Rootletin-depleted mouse photoreceptors show signs of degeneration at 18 months, reflected by shortening, disorganisation and loss of the photoreceptor outer segments [6]. The requirement for the rootlet was interpreted as the need for the small connecting cilium to hold in place the large outer segment [3]. It is notable that cells with less prominent rootlets did not show this phenotype.

Thus, the hypothesis has emerged that the rootlet is required in the stability and function of cilia that are subjected to mechanical stress. Other functions for the rootlet in cilium biology, particularly in cells other than photoreceptors, are unclear. An association with KLC-3 led to suggestions that it might be involved in transport to the cilium or in facilitating intraflagellar transport (IFT) at the base of the cilium, but no transport defect was noted in rootletin mutant mice [6]. However, in *Caenorhabditis elegans* the rootletin orthologue, *Che-10*, was shown to indirectly influence IFT by modulating the preassembly/localisation of various IFT proteins to the periciliary membrane compartment [8]. Interestingly, in *Che-10* mutants, cilia are initially formed normally but start to degenerate in late larvae.

Aside from cilia, rootletin has also been shown to have a role in centriole cohesion during the centrosome duplication cycle [7, 9–11]. In metaphase cells, the centrosome consists of two tightly associated centrioles. After mitotic (M) phase exit, but before cell division, these become separated but loosely attached via linker proteins, known as a G1-G2 tether [11]. Rootletin has been shown to be one of these linker proteins and so is required for centriole cohesion in G1 and S phases. It associates with the related C-Nap1, which is itself associated with the ends of centrioles, thereby forming filaments that maintain a loose connection between the centrioles [7, 9]. Phosphorylation of C-Nap1/rootletin by Nek2 kinase allows separation of the centrioles in late G2 before proceeding to M phase [9].

Here, we explore the function of the presumed *Drosophila* homologue of rootletin, which is encoded by the gene *CG6129* (hereafter referred to as *Rootletin*) [4, 12]. *Drosophila* displays several distinctive features relevant to *Rootletin* function. First, the centrosome duplication cycle is modified in *Drosophila* such that there is no G1-G2 tether either in the early embryo [13] or larval neuroblasts [14]. Instead, the centrosomes split immediately after mitosis. It is therefore of interest to ask whether *Rootletin* is required for *Drosophila* centrosome duplication.

**Fig. 1:**
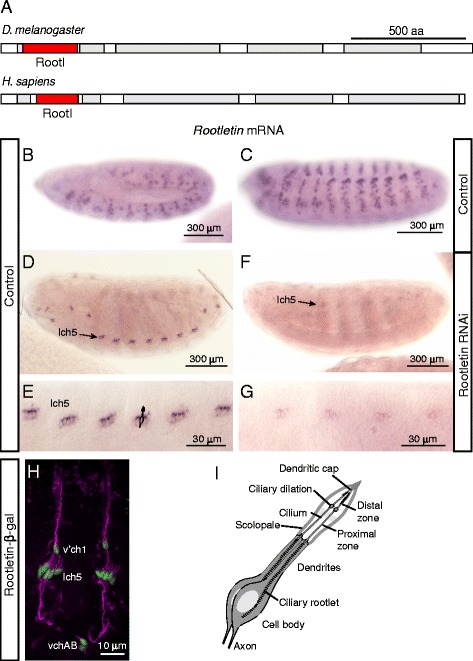
*Rootletin* expression pattern and knock-down efficiency. **a** Schematic of protein structure of *Drosophila* and human Rootletins. Regions of high probability of coiled-coil (grey) were found by MARCOIL (http://toolkit.tuebingen.mpg.de/marcoil). The ‘rootletin’ domain is shown in *red*. **b**–**g** *Rootletin* mRNA in embryos. **b** Wild type, stage 12. **c** Wild type, stage 14. **d** Wild type, stage 17, lch5 indicates lateral Ch organ clusters. **e** Higher magnification view of (**d**). **e** *Rootletin* knock-down, stage 17. **g** Higher magnification view of (**f**). **h** Wild-type stage 17 embryo of a *Rootletin-lacZ* enhancer reporter fusion, showing β-galactosidase immunoreactivity in Ch neurons (*green*), counterstained for sensory neurons with anti-Futsch antibody (*magenta*). Lch5, v’ch1 and vchAB are the designations of the abdominal Ch neurons. **i** Schematic representation of the *Drosophila* Ch organ structure with sensory cilia at the tips of the neuronal dendrites housed in a scolopale structure

A second feature of *Drosophila* is that it has very few ciliated cell types. The only somatic cells bearing cilia are the type I sensory neurons, in which olfactory, gustatory or mechanosensory reception are performed via a specialised terminal cilium [15]. Whilst the cilia in these classes of neuron all have an associated rootlet, the most robust and prominent rootlets are found in the chordotonal (Ch) neurons [16, 17] (Fig. 1i). Ch neurons are auditory and proprioceptive mechanosensors and may be presumed to be under mechanical stress. Although nothing has been described of *Rootletin* function, it is highly represented in the transcriptome of Ch neurons [18]. We investigated the expression and function of *Drosophila Rootletin* with particular focus on Ch neuron structure and function.

## Methods

### Fly stocks

The RNA interference (RNAi) knock-down stocks, *w*^*1118*^*; P{GD11829}v22694, P{KK102209}VIE­260B,* and *y w;* and *P{attP,y[+],w[3′]}* lines were obtained from Vienna *Drosophila* Resource Center. Most experiments were carried out using the first RNAi line, but a number were also replicated using the second line (notably the behavioural experiments and the Sas-4 localisation). The *w*^*1118*^, UAS­*mCD8­GFP* and UAS­*Dcr2* stocks were obtained from Bloomington Stock Center (Indiana University, Bloomington, IN, USA). Other stocks used were *Rootletin­GFP* (a gift from Elisabeth Cortier and Benedicte Durand [12]), *bam­Gal4:VP16* (a gift from Helen White­Cooper), *sca­Gal4* and *RempA­YFP* (a gift from Maurice Kernan). In all experiments, control animals were obtained by crossing the appropriate Gal4 driver line to *w*^*1118*^ (control for the GD RNAi) or *y w; P{attP,y[+],w[3′]* (KK RNAi control).

### Immunohistochemistry

Primary antibodies used were mAb­Futsch (1:200), RbAb­HRP (1:500), RbAb­GFP (1:500) (Molecular Probes), mAb­NompC (a gift from Jonathon Howard) and RbAb­Sas4 (a gift from Jordan Raff). All secondary antibodies were obtained from Molecular Probes. For immunofluorescence microscopy, embryos were observed using a Zeiss Pascal confocal microscope. Images were processed for contrast and size in ImageJ.

### In situ hybridisation

mRNA in situ hybridizations to whole embryos were carried out by standard digoxigenin method. Gene fragments were amplified from genomic DNA by PCR and digoxigenin­labelled RNA probes prepared using T7 RNA polymerase (primers used: 3′GTAATACGACTCACTATAGGGTTGTAGGGCCATTTGTAGCC, 5′GAAGGCTCAAGTGGAGTTCG). For bright­field microscopy, samples were observed using an Olympus AX70 microscope and captured with a DP50 camera.

### Promoter fusions

A 406-bp fragment from the first intron of CG6129 was amplified from genomic DNA using primers 3′TCTAGACTCGCCTACGCTACAGACG and 5′GAATTCACGAGGACAAGGAAATTATTATGA. The fragment was cloned into pLacZ­attB β­gal reporter plasmid. Transformants were made by microinjection into syncytial blastoderm embryos. The injection line used carries the attP landing site at locus 68E.

### Electron microscopy

Whole adult heads were removed and rinsed in 0.5 % Triton X­100. The proboscis was removed to facilitate infiltration of the fix, and the heads were then fixed in 2.5 % glutaraldehyde and 2 % paraformaldehyde in 0.1 M phosphate buffer (pH 7.4) overnight at 4 °C. Heads were then washed in 0.1 M phosphate buffer (pH 7.4), postfixed with OsO4, dehydrated in an ethanol series and embedded in Polybed812. Ultrathin (75 nm) sections of the antennae were then stained with aqueous uranyl­acetate and lead citrate and examined with a Hitachi 7000 electron microscope (Electron Microscopy Research Services, Newcastle University Medical School).

### Adult climbing assay

Fifteen mated young female flies were placed in a 100-ml measuring cylinder and knocked down to the bottom of the cylinder. After 10 s, the distance ascended by each fly was measured. At least three biological replicates of 15 flies were performed.

### Larval crawling assay

Five third instar larvae were placed on a 2 % agar gel in a 24-cm^2^ plate, and their crawl paths were traced for 2 min. The traces were measured using NeuronJ plug in to ImageJ open source software.

### Larval hearing assay

Five third instar larvae were placed on an agar plate. The plate was placed directly on top of a speaker and a pure 1000-Hz tone of 1-s duration was played three times, 30 s apart at a volume of 92 dB. A retraction response during the tone was ranked as ‘1’ and no response as ‘0’. Each replica result is presented as an average of the number of larvae responding to sound during each of the three tones played. Overall result is presented as an average of averages of the number of larvae responding to the tone. The experiment was repeated in eight replicas for the sample and the control.

### Adult grooming assay

The procedure used was modified from that described by Vandervorst and Ghysen [19]. Flies were decapitated to prevent voluntary movement, but maintaining their reflex responses such as grooming. Individual thoracic mechanosensory bristles were deflected with a pin, and the response recorded (in wild-type flies a grooming response results whereby the fly raises one of its legs to clean the area of the bristle deflected).

## Results

### In the embryo, *Drosophila Rootletin* is expressed exclusively in developing ciliated neuron lineages

*CG6129* encodes a predicted protein of 2048 amino acid residues. The amino acid sequence has 24 % identity and 58 % similarity to that of human rootletin (encoded by the *CROCC* gene) and has a similar domain structure [4] (Fig. 1a); on this basis, *CG6129* has been annotated as *Rootletin* in FlyBase [20]. The *Drosophila* Rootletin secondary structure as predicted by ModBase [21] indicates a globular head domain and coiled-coil structured tail domain. There are no other *Drosophila* homologues similar to CROCC, or indeed to the related C-Nap1 (CEP250).

In situ hybridisation of embryos shows that *Rootletin* mRNA is not expressed ubiquitously but in a dynamic pattern corresponding exclusively to the developing sensory neuron lineages (Fig. 1). Expression begins at stage 12 in a pattern that suggests it is expressed in the precursors and progeny cells of type I sensory lineages of all sensory modalities, including olfactory, external sensory (ES—mechanosensory and gustatory) and Ch (auditory and proprioceptive) neurons (Fig. 1b). Expression continues in these lineages during their asymmetric cell divisions and early terminal neuronal differentiation, during which time the sensory cilium is generated (Fig. 1c). However, by stage 15, *Rootletin* mRNA becomes reduced in most of lineages but remains strong in a subset of differentiating neurons (Fig. 1d, e). The location of these cells is consistent with them being the Ch neurons. This is consistent with the previous observation that *Rootletin* is highly enriched in the transcriptome of developing Ch neurons [18]. Moreover, a β-galactosidase fusion gene driven by part of the *Rootletin* first intron is expressed exclusively in late differentiating Ch neurons (Fig. 1h). Given the mRNA pattern, it seems likely that this reporter construct contain a Ch-specific enhancer for this late component of Rootletin expression.

We conclude that *Rootletin* is expressed exclusively and transiently in the lineages leading to cells that differentiate a cilium, i.e. the type I sensory neurons in the body wall. However, it is persistent in the differentiating Ch neurons, which are the cells with the most robust rootlet. Rootletin appears not to be expressed in other cells of the embryo. We cannot rule out very low level basal expression in other cells, but this seems unlikely given the high ranking in the previous transcriptomic analysis [18].

### *Rootletin* knock-down in sensory lineages does not affect cell divisions

The lack of general expression suggests that *Rootletin* does not play a role in cell divisions. However, the mRNA is present in dividing sensory cells. Therefore, it is possible that Rootletin plays a role in cell division within these lineages. Type I sensory neurons arise from individual sense organ precursor cells. Each precursor divides asymmetrically several times to give the sensory neuron and 3–4 support cells of a single sense organ [15]. We assessed the function of *Rootletin* by RNA interference using a fly stock containing a Gal4-inducible hairpin RNA construct for the gene, referred to as UAS-*Rootletin*-RNAi. Line *P{GD22694}v22694* was used for the majority of experiments, but a second independent line, *P{KK102209}VIE­260B*, was also used where stated below. RNAi was induced using a *scaGal4* driver, which is expressed in neurectoderm and developing neural cells. *scaGal4, UAS-Rootletin-*RNAi embryos exhibited a large reduction in *Rootletin* mRNA (Fig. 1f,g).

**Fig. 2:**
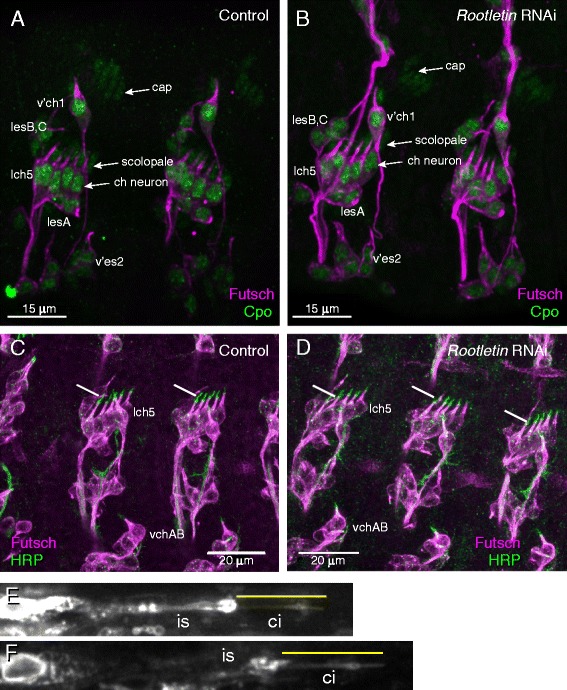
*Rootletin* knock-down does not affect Ch lineage cell divisions or cilium formation. **a**, **b** Stage 17 embryos stained with anti-CPO (*green*) and anti-Futsch (*magenta*). **a** Wild type, showing lch5 and v’ch1 Ch neurons. Three cell types found in the chordotonal organ are indicated (*neuron*, *scolopale*, *cap*). Also indicated are ES neurons (*lesA–C*, *v’es2*). **b** *Rootletin* knock-down, all these cell types are visible. **c**, **d** Stage 16 embryos stained with anti-Futsch (magenta) and anti-HRP (*green*). **c** Wild type, showing the HRP-positive cilia at the tips of the Ch neurons (lch5 and vchAB). **d** *Rootletin* knock-down, showing that the ch neuron cilia are present. **e**, **f** Cilia of adult femoral Ch neurons detected with mCD8-GFP. The terminal dendrite of single neurons is shown with the non-ciliary inner segment (*is*) and cilium (*ci*, *yellow line*) indicated. **e** Wild type. Cilium length = 9.5 μm (**f**) *Rootletin* knock-down., Cilium length = 9.8 μm

If *Rootletin* knock-down causes cell division disruption, this would be expected to affect the numbers and/or types of cells being formed in the sense organ lineages. However, there was no indication of a cell division defect in the sensory neuron lineages: *Rootletin* knock-down flies were viable with normal numbers and arrangements of bristles (produced by support cells of ES lineages) (data not shown). In order to visualise all cell types of the sense organ lineages, late embryos were stained with antibodies against Couch Potato, a protein expressed in all sense organ precursors and their progeny [22], and Futsch, a protein expressed in sensory neurons [23]. No differences were observed between knock-down embryos and controls (Fig. 2a, b). For instance, each chordotonal organ of the lateral lch5 cluster consisted of a single neuron and several supporting cells in their correct locations. Moreover, staining dividing sense organ precursor cells with γ-tubulin showed normal centrosomes and mitotic spindle (data not shown). Therefore, we found no evidence of any phenotypes that might indicate cell division defects resulting from centrosome cycle disruption.

### *Rootletin* knock-down in Ch neurons does not strongly affect cilium formation but causes loss of ciliary rootlet

We looked for the presence of the Ch neuron cilia initially by immunohistochemistry using anti-horse radish peroxidase (HRP). In the embryo, this antibody detects an epitope that is transported along the newly formed cilium of differentiating Ch neurons (Fig. 2c). In *Rootletin* knock-down embryos, anti-HRP immunoreactivity appeared normal, showing that Ch neuron cilia were present (Fig. 2d). To assess the mature Ch cilium itself, we examined the femoral Ch neuron array in adult flies expressing the mCD8-GFP marker in sensory neurons (*scaGal4, UAS-mCD8-GFP* flies). We could not detect any consistent change in ciliary morphology (Fig. 2e, f) or length (control: mean cilium length = 9.46 μm, sd = 1.18; RNAi: mean = 9.79, sd = 1.01, *n* = 15).

**Fig. 3:**
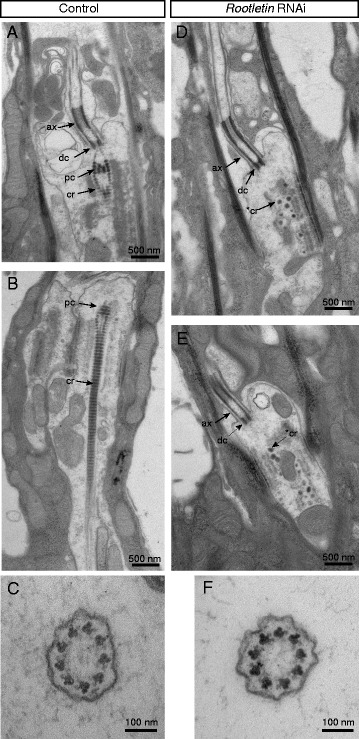
*Rootletin* knock-down results in loss of rootlet and proximal centriole. Transmission electron microscopy of Ch neuron dendrites in the adult antenna (Johnston’s organ). **a**–**c** Wild type. **a** Longitudinal section of cilium base showing the proximal centriole (*black arrow*, *pc*) and a robust ciliary rootlet structure (*black arrow*, *cr*). **b** Longitudinal section of cilium showing the axoneme (*ax*), distal centriole (*dc*), proximal centriole (*pc*), and ciliary rootlet (*cr*). **c** Transverse section of cilium at a level proximal to the ciliary dilation. Regular axonemal ninefold symmetry is visible. **d**–**f** *Rootletin* knock-down. **d**, **e** Two examples of longitudinal sections labelled as before. Note the lack of ciliary rootlet structure and the proximal centriole. Instead, electron-dense aggregates are visible, possibly representing remains of the ciliary rootlet (*cr). **f** Transverse section at a level proximally to the ciliary dilation. Regular axonemal ninefold symmetry is apparent

To determine the effect of *Rootletin* knock-down on the cilium in more detail, we used transmission electron microscopy (TEM) to examine the large array of Ch neurons (Johnston’s organ) in the adult second antennal segment which is required for proprioception, gravitaxis and auditory sensation [24]. In longitudinal sections of the Ch organ units (scolopidia) of control antennae, the cilia of the sensory neurons and their prominent striated rootlets are readily visible (*n* = 27 cilia, Fig. 3a, b). In *Rootletin* knock-down antennae, the cilia themselves appeared generally normal and features such as the ciliary dilation were visible. In transverse sections, the 9 + 0 microtubular structure of the axoneme appeared normal (Fig. 3c, f). In contrast, the ciliary rootlet was completely disrupted and appeared to be absent in all cilia sectioned from knock-down flies (*n* = 23, Fig. 3d, e). In many instances, electron-dense ‘globules’ could be observed where the rootlet should be.

**Fig. 4:**
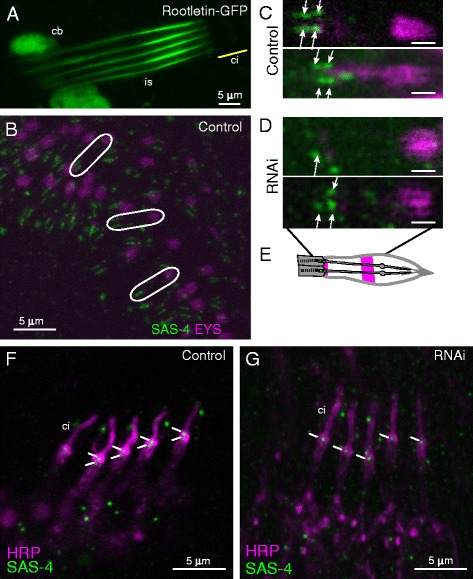
*Rootletin* knock-down disrupts basal body structure. **a** Ch neurons (*lch5*) of unfixed third instar larva transgenic for Rootletin-GFP partial fusion protein showing strong localisation to the inner segment (*is*) extending from the base of the cilium (*ci*, approximate location shown by *yellow line*). Some expression is also observed in the cell bodies (*cb*). **b**–**d** Ch neurons of pupal antenna visualised with anti-Eys (*magenta*) and anti-Sas-4 (*green*) antibodies. **b** Wild type at low magnification. *White ellipses* indicate single chordotonal organs, each with two neurons, with two pairs of Sas-4 staining puncta representing the centrioles. Eys is mainly seen in a distal band surrounding the cilium but also at the base of the cilium (a pattern similar to anti-HRP). **c** High magnification view of two examples, *white arrows* indicate two pairs of Sas-4 puncta representing two centrioles for each neuron. Scale bar = 1 μm. **d** *Rootletin* knock-down. Images show two possible phenotypes of either just one Sas-4 punctum or two Sas-4 puncta missing from each chordotonal organ. Scale bar = 1 μm. **e** Schematic representation of the Sas-4 and Eys protein localisation within the Ch organs in wild-type antenna. **f**, **g** Immunofluorescence analysis of stage 17 embryonic Ch neurons (lch5 cluster) visualised with anti-HRP (*magenta*) and anti-Sas-4 (*green*). *White lines* indicate the positions of Sas-4 staining associated with the centrioles. **f** Wild type. **g** *Rootletin* knock-down

The loss of the rootlet is consistent with Rootletin being a major rootlet component. Indeed, a fusion protein consisting of the N-terminal region of Rootletin fused to green fluorescence protein (GFP) was previously shown to localise to a rod-like structure in embryonic Ch neurons [12]. We confirm here in mature larval Ch neurons that this fusion protein delineates a rod-like structure extending from the base of the cilium through the entire inner segment of the dendrite (Fig. 4a).

In summary, Rootletin knock-down appears to have no gross effect in ciliogenesis but causes loss of the rootlet. Thus, the presence of Rootletin as a major component of the ciliary rootlet is conserved in *Drosophila* sensory neurons.

### *Rootletin* knock-down results in dissociation of the proximal and distal centrioles of Ch neuron cilia

In addition to loss of the rootlet, TEM revealed an effect on the proximal centriole of the basal body. This structure is normally surrounded by strands of the rootlet leading down from the distal centriole (Fig. 3b). In TEM sections from Rootletin-depleted cells in which the base of the cilium was visible, the proximal centriole could not be observed (Fig. 3d, e).

We then examined the basal body by immunofluorescence using an antibody to the Sas-4 protein, which is located at the distal ends of the centrioles in late embryos [14]. We examined the developing Ch neurons of Johnston’s Organ in the pupal antenna. In this tissue, most scolopidia house two Ch neurons, and these are associated with a pair of Sas-4 puncta in the tip of the inner dendritic segment of Ch neurons (Fig. 4b–d). These correspond to the proximal and distal centrioles. In *Rootletin* knock-down pupae, a proportion of Ch neurons showed only the distal punctum, suggesting that the proximal centriole is missing from the base of the cilium (Fig. 4d) (out of 34 neurons, 27 showed a single punctum). However, due to the punctate background given by this antibody, it is not possible to say whether the proximal centriole punctum is entirely missing or simply mislocated within the cell. This phenotype was also observed in the embryonic Ch neurons. At stage 16/17 of embryogenesis, lch5 neurons are associated with two Sas-4 puncta (Fig. 4f). In Rootletin-depleted cells, frequent loss of the proximal punctum was observed (Fig. 4g). The loss of the proximal punctum staining was observed in both Rootletin RNAi lines.

### *Rootletin* knock-down results in uncoordinated and auditory phenotypes indicating defective Ch neurons

**Fig. 5:**
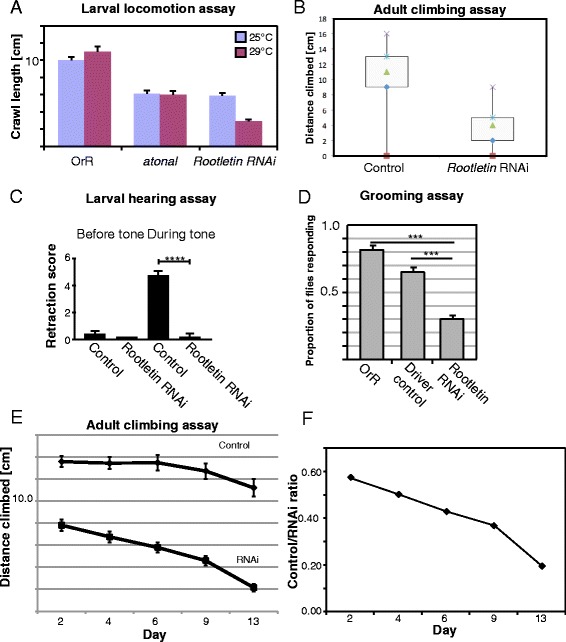
*Rootletin* knock-down severely disrupts Ch neuron function. **a** Larval locomotion assay showing that *Rootletin* knock-down larvae move less due to uncoordination. Larvae were tested at two temperatures as the Gal4 driving the RNAi construct is more active at higher temperatures (RNAi predicted to be more complete). Larvae from an *atonal* mutant stock were tested for comparison; such larvae lack Ch neurons. **b** Adult climbing assay show a significant disruption of climbing behaviour in adult *Rootletin* knock-down flies. Results plotted are averages of three trials of 15 flies. **c** Larval hearing assay. Larvae are tested in groups of five and the retraction score refers to the average number of larvae that contract. *n* = 40, *error bars* represent standard deviation. **d** Grooming assay for behaviour in response to sensory bristle deflection. Proportion represents an average response proportion from six replicas each testing five flies. *Error bars* represent standard error. **e** Adult climbing assay performed ageing flies. Results are plotted as averages from four replicas of 15 flies; the same flies were used in each time point, *error bars* represent standard error, two-way Anova *p* > 0.003, **f** Plot of ratio of wild-type/RNAi knockdown for data shown in (**e**). The rate of performance decrease over time is greater for *Rootletin* knock-down flies

To test whether the loss of *Rootletin* and the rootlet affects Ch sensory neuron function, we analysed behaviour of larvae and adults. Mutations that affect Ch neuron function cause impairments of larval and adult locomotory behaviours due to their proprioceptive function [25]. Compared with control larvae, knock-down larvae show reduced crawling path lengths (Fig. 5a). The reduction is at least as severe as that exhibited by *atonal* mutant larvae, which lack Ch neurons. Similarly, knock-down adult flies show impaired behaviour in a climbing assay (Fig. 5b). This phenotype was strengthened by greater Gal4 activity (higher incubation temperature) and by enhancement of RNAi with UAS-Dcr-2 and was observed for both Rootletin RNAi lines. Given that *Rootletin* expression is confined to sensory cells, the results are consistent with defective sensory neuron function.

*Drosophila* third instar larvae are capable of detecting substrate-borne vibrations (‘hearing’), and momentarily retract in response to a 1-kHz tone. This behaviour is mediated by the Ch neurons [26]. In contrast to control larvae, *Rootletin* knock-down larvae completely lost the ability to respond to tones, suggesting an absence of auditory function of the Ch neurons (Fig. 5c). Overall, it seems that despite the presence of a grossly normal cilium, the Ch neurons appear functionally to be highly defective.

ES neurons have a small ciliary rootlet [27], and *Rootletin* is expressed transiently in ES neurons during their early differentiation. Although they fail the climbing assay, *Rootletin* knock-down flies are otherwise relatively normal in posture and movement. This suggests that ES neurons that innervate the mechanosensory bristles of the fly are not likely to be strongly affected. However, it is notable that at higher Gal4 activity levels, the adult climbing impairment becomes more severe than that of *atonal* mutants, which lack Ch neurons but have normal ES neurons (Fig. 5a). This suggests that there is an effect on both Ch neurons at ES neurons at this level of knock-down. To test whether adult ES organs (sensory bristles) were functional, we carried out a grooming assay. Experimental deflection of mechanosensory bristles on the thorax of a control fly results in a grooming response—a leg is raised to clean the area of the bristle. For *Rootletin* knock-down flies, we found that this response was induced less frequently (Fig. 5d). We suggest this is evidence for partially defective mechanosensory response of ES neurons.

By analogy to the observations on mouse photoreceptors, the lack of rootlet might render the Ch neurons more susceptible to mechanical stress and thus perhaps their function may deteriorate further with age. We assessed Ch neuron function as the flies age using climbing assay performance. In this assay, we observed a decline in performance with time that occurs at a faster rate than for control flies, suggesting some age-dependent deterioration in Ch neuron function (Fig. 5e, f). To assess Ch cilium morphology over time, we examined the femoral Ch neuron array in flies expressing the mCD8-GFP marker in sensory neurons (*scaGal4, UAS-mCD8-GFP* flies). At this level of observation, we could detect no change in ciliary morphology, or consistent change in length in the ageing flies (data not shown).

### Effect of Rootletin knock-down on ciliary proteins

**Fig. 6:**
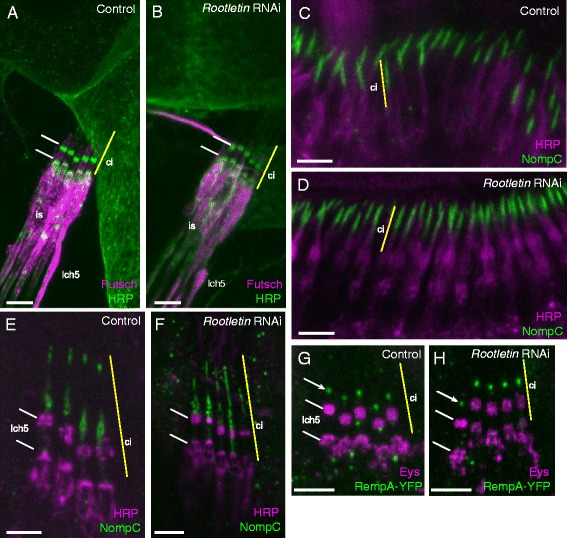
Transport of proteins in *Rootletin* knock-down Ch cilia. **a**, **b** Anti-HRP immunoreactivity (*green*) in Ch neurons of mature larvae, counterstained with anti-Futsch (*magenta*). **a** Wild type, two bands of anti-HRP staining are visible at the cilium base and surrounding a medial point in the cilium (*white lines*). *Yellow line* indicates approximate extent of the cilium (*ci*) at the tip of the dendrite inner segment (*is*). **b** The same bands are visible in *Rootletin* knock-down. **c**, **d** NompC localisation (*green*) in pupal Ch neuron cilia of Johnston’s organ; counterstained with anti-HRP (*magenta*). **c** Wild type. **d** *Rootletin* knock-down. **e**, **f** NompC localisation (*green*) in mature larval Ch neurons (*lch5*), counterstained with anti-HRP (*magenta*) (HRP bands indicated by *white lines*). **e** Wild type, the NompC is localised distally to the HRP immunoreactivity. **f** *Rootletin* knock-down, NompC is grossly normal, but shows some mislocalisation to the proximal cilium in one of the neurons. **g**, **h** RempA-YFP localisation (*green*) in mature larval Ch neurons (*lch5*), counterstained with anti-EYS (*magenta*) (HRP bands indicated by *white lines*). **g** Wild type, showing puncta of localisation at the ciliary dilation (*white arrow*). **h** *Rootletin* knock-down, showing no change in RempA-YFP localisation. All scale bars = 5 μm

Although no gross defect in cilium structure was detected above, Ch neurons appear to be severely defective in function when *Rootletin* is reduced. One possibility is that mechanotransduction proteins are defectively transported and/or localised in the cilium. To test this, we initially examined localisation of the epitope detected by anti-HRP. As stated above, this is initially transported along the embryonic Ch neuron cilium, then at late embryonic stages, it appears to be secreted into the luminal space surrounding the cilium and becomes refined to two bands, one at the cilium base and the other at a medial location along the cilium. These are visible in Ch neurons of mature larvae (Fig. 6a). In *Rootletin* knock-down larvae, there appeared to be no alteration to this banding pattern (Fig. 6b).

As a further test of protein localisation, we examined NompC. NompC is a mechanosensory channel protein that is transported into the Ch neuron cilium by IFT and then localises specifically in the distal part of the cilium [28, 29]. In the developing Ch neurons of the pupal antenna, NompC was detected in the tip of the cilia (Fig. 6c). This localisation appeared unaffected by Rootletin knock-down (Fig. 6d). In the mature Ch neurons of the wild-type larva, NompC is detected at the ciliary dilation and the tip of the cilium (Fig. 6e). In *Rootletin* knock-down larvae, NompC was detected within the cilium as normal, but in some cases its localisation appeared to spread into the proximal region of the cilium (Fig. 6f). The average extent of NompC localisation in knock-down cilia was 11.1 μm compared to 8.7 μm in control cilia (*p* = 0.0025, *n* = 8).

These data suggest that NompC transport into the cilium is largely normal, suggesting no gross defect in transport. However, there may be a partial defect in specific localisation to zones. Delineation of the specialised ciliary zones has been shown to require the gene, *rempA* [29], which encodes the orthologue of the IFT-A protein IFT140, part of the retrograde IFT machinery. RempA-YFP fusion protein localises to the ciliary dilation in mature larval Ch neuron cilia (Fig. 6g) [30]. This localisation did not appear to be altered in *Rootletin* knock-down larvae (Fig. 6h).

## Discussion

*Drosophila Rootletin* (*CG6129*) is required for the formation of the ciliary rootlet of Ch neurons. This strongly supports the conclusion that it performs a conserved function as a structural component of the rootlet. The expression pattern of *Rootletin* also highlights this function: it is restricted to ciliated cells, i.e. the type I sensory neurons, and among these cells, it is most abundantly and persistently expressed in Ch neurons, whose cilia have very robust rootlets. In contrast, *Rootletin* does not seem to be required for non-ciliated cells.

In Ch neurons, loss of *Rootletin* and the rootlet results in functionally defective sensory responses. Despite this, the Ch neuron cilium itself does not appear to be strongly defective structurally. This may suggest a defective mechanotransduction process rather than defective development of the cilium. Based on observations in mouse photoreceptors, it was proposed that a rootlet is required for mechanical stability of large or motile ciliary structures [2, 3, 6]. Clearly, the Ch neurons could be described in this category as these mechanosensory cells must be under mechanical stress in their function. Moreover, the Ch neuron cilium has the molecular machinery for ciliary motility, and there is biophysical evidence that cell or ciliary motility might be important for mechanotransduction [24]. The rootlet can therefore be seen to provide a solid anchor for this in order to maintain dendritic integrity. Age-related decline in fly proprioceptive function may be consistent with the stress/anchor hypothesis. However, at the light microscope level, we found no sign of collapse or shortening of the cilium with age in *Rootletin* knock-down flies.

An alternative explanation for Ch neuron dysfunction and age-related decline is a requirement for rootlet/*Rootletin* in transport of components to the base of the cilium or within the cilium, as has been proposed for the orthologue in *C. elegans* [8]. In *Drosophila* Ch neurons, it is clear that transport and IFT are not strongly defective as ciliogenesis appears to occur largely normally. Whilst subtle changes in the channel NompC localisation suggest that *Rootletin* might be indirectly involved in some aspects of IFT, no change in the localisation of IFT protein, RempA, suggests a lack of general disruption of IFT. It is possible, however, that a subtle impairment of IFT disrupts transport necessary for long-term ciliary homeostasis rather than ciliogenesis. Given the severe loss in neuronal function, even in young flies, an alternative explanation is that the rootlet directly participates in mechanotransduction, such as being required to maintain or transmit tension during cilium stimulation.

In other organisms, rootletin is required for centriole cohesion or tethering after centriole duplication [7, 31]. In vertebrates, rootletin forms the tether in association with the related C-Nap1 (CEP250) protein [7]. It seems unlikely that Rootletin is the centrosome linker protein in *Drosophila* because it is not expressed generally. Moreover, there is no separate C-Nap1 orthologue in *Drosophila*. Lack of this function would be consistent with observations that *Drosophila* cells seem to lack centriole tethering. Instead, centrioles separate immediately upon disengagement during the centrosome cycle [32]. In mammalian cells, centrosome separation upon entering mitosis is achieved by Nek2 phosphorylation of rootletin and C-Nap1 [30]. Interestingly, *Drosophila* retains an orthologue of Nek2, and in cultured *Drosophila* cells Nek2 knockdown causes mitotic spindle defects [33]. In the absence of a role for Rootletin or C-Nap1, the role of Nek2 in this process is unclear.

Despite the lack of a role in centriole tethering in centrosomes, it seems that Rootletin plays a ‘tethering-like’ role in the basal body of the Ch neuron cilium since the proximal centriole is lost upon *Rootletin* knock-down. Indeed, on TEM of wild-type neurons, the proximal centriole appears to be held in place by strands of the rootlet that pass around it before joining with the distal centriole.

## Conclusions

The role of Rootletin as a component of the ciliary rootlet is conserved in *Drosophila*. In contrast, lack of a general role in cell division is consistent with lack of centriole tethering during the centrosome cycle in *Drosophila*. Although our evidence is consistent with an anchoring role for the rootlet, severe loss of mechanosensory function of Ch neurons upon *Rootletin* knock-down may suggest a direct role for the rootlet in the mechanotransduction mechanism itself. In contrast, any effect on ciliary transport appears to be subtle.

## Abbreviations

Ch: chordotonal
ES: external sensory
GFP: green fluorescence protein
HRP: horse radish peroxidase
IFT: intraflagellar transport
KLC-3: kinesin light chain 3
RNAi: RNA interference
TEM: transmission electron microscopy
